# CHILDHOOD CONDITIONS AND ARTHRITIS AMONG MIDDLE-AGED AND OLDER ADULTS IN CHINA: THE MODERATING ROLES OF GENDER

**DOI:** 10.1093/geroni/igz038.1020

**Published:** 2019-11-08

**Authors:** Nan Lu, Bei Wu, Nan Jiang, Tingyue Dong

**Affiliations:** 1 Renmin University of China, Beijing, China; 2 New York University, New York, New York, United States; 3 Columbia University, New York, United States

## Abstract

This study examined the moderating effect of gender on the relationship between childhood conditions and arthritis among middle-aged and older adults in China. The data were derived from the 2015 wave and the life history module of the China Health and Retirement Longitudinal study. The sample included 19800 respondents age 45 and over. Multiple imputation was used to handle the missing data. A multilevel logistic regression was used to test the proposed models. Childhood socioeconomic status, mother’s education, subjective health, access to health care and medical catastrophic event were found to be significant factors associated with arthritis in later life, after controlling for adulthood and older age conditions. Furthermore, the effects of childhood socioeconomic status on arthritis were found to be higher among men than women. The findings highlight the important role of childhood conditions that affect the onset of arthritis in later life. Policy and intervention implications are discussed.

